# Contingencies and attentional capture: the importance of matching stimulus informativeness in the item-specific proportion congruent task

**DOI:** 10.3389/fpsyg.2014.00540

**Published:** 2014-06-02

**Authors:** James R. Schmidt

**Affiliations:** Department of Experimental Clinical and Health Psychology, Ghent UniversityGhent, Belgium

**Keywords:** conflict adaptation, attention, contingency learning, attentional capture, proportion congruent, ISPC, informativeness, Stroop

The proportion congruent effect is the observation that congruency effects are smaller when the proportion of incongruent stimuli is higher. The conflict adaptation account argues that this effect is due to a shift of attention away from the source of conflict. In contrast, the contingency account proposes that participants learn to predict the likely response on the basis of the distracter, and this produces a proportion congruent effect incidentally. However, some have argued that conflict adaptation can be observed in the restricted scenarios in which the mostly incongruent stimuli are not strongly predictive of the correct response. This opinion article argues that comparing predictive to non-predictive stimuli might be problematic. Some learning research would suggest that attention to the distracter should vary, but for an entirely different reason than that proposed by conflict adaptation theory: contingent stimuli attract attention.

In the Stroop paradigm (Stroop, 1935), participants are tasked with the goal to ignore a distracting color word and to respond to the print color it is presented in. Only partially successful at doing so, participants respond slower and less accurately to incongruent stimuli (e.g., the word “blue” printed in red) than to congruent stimuli (e.g., “blue” in blue). Similar congruency effects are observed in the Simon (Simon and Rudell, 1967), flanker (Eriksen and Eriksen, 1974), picture-word (Rosinski et al., 1975), and various other comparable tasks. Such congruency effects are reduced if the relative number of incongruent trials is increased (Lowe and Mitterer, 1982). This *proportion congruent (PC) effect* is most commonly interpreted as evidence for the *conflict adaptation account* (e.g., Botvinick et al., 2001). This account argues that detection of conflict results in a decrease of attention to the source of conflict (e.g., the word in a Stroop task). Because conflict is more frequent in a mostly incongruent condition, attention to the word is particularly low. The word therefore has little impact on performance, and the congruency effect is resultantly small.

Though seemingly intuitive, the conflict adaptation account does face some important challenges. For instance, consider the item-specific PC effect (Jacoby et al., 2003). In this variant of the PC task, some words are mostly congruent and others are mostly incongruent. These two item types are intermixed into one procedure, but there is nevertheless a smaller effect for mostly incongruent items. This might be described in terms of item-specific adaptations of attention (e.g., Blais et al., 2007), though this requires the unintuitive assumption that attention to the word is determined by the identity of the word (which must, of course, first be identified; but see Verguts and Notebaert, 2008). Alternatively, the *contingency account* proposes that the entire item-specific PC effect is explained by the learning of contingent relationships between distracting words and responses (Schmidt and Besner, 2008; Schmidt, 2013b; for a review, see Schmidt, 2013a). For mostly congruent stimuli, each word is presented most often with the congruent color. This means that, for instance, the word “blue” is predictive of a blue response. Congruent trials thus benefit from this prediction, and the congruency effect increases. For mostly incongruent stimuli, the reverse is true: each word is presented most often with a specific incongruent response. Thus, “red” might be predictive of a yellow response. Incongruent trials thus benefit, and the congruency effect decreases. Some evidence argues compellingly in favor of the contingency account (Schmidt and Besner, 2008; Atalay and Misirlisoy, 2012; Grandjean et al., 2013; Schmidt, 2013b). For instance, Schmidt (2013b) presents a dissociation procedure in which contingency learning and conflict adaptation could be separately assessed. Specifically, it was possible to compare sets of incongruent trials that were: (a) equivalent in PC (mostly incongruent) but that varied in contingency (high vs. low), or (b) equivalent in contingency (low contingency) but that varied in PC (mostly congruent vs. mostly incongruent). Thus, the former set allows an assessment of contingency learning in the absence of conflict adaptation, and the latter set allows an assessment of conflict adaptation in the absence of a contingency bias. These comparisons revealed a very strong contingency effect, with no evidence for conflict adaptation. The (item-specific) PC effect thus might have nothing to do with conflict adaptation at all. Some neuropsychological data even argues that the area claimed to be involved in (item-specific) conflict adaptation (viz., the anterior cingulate cortex; see Blais and Bunge, 2010) might instead be involved in contingency learning (Grandjean et al., 2013).

On the other hand, many argue that conflict adaptation can be observed independently of contingency biases (e.g., Crump and Milliken, 2009; Blais and Bunge, 2010; Bugg et al., 2011; Abrahamse et al., 2013; Bugg and Hutchison, 2013). One specific claim is that, while contingency learning might dominate performance in some scenarios, conflict adaptation might still be observable in others (Bugg et al., 2011; Bugg and Hutchison, 2013). For instance, Bugg and colleagues argue that if the target is easier to process than the distracter, use of contingencies associated with the distracter might be impaired. The target then might serve as a cue to PC. Of interest for the current discussion, Bugg and Hutchison further argue that in designs where mostly congruent and mostly incongruent stimuli are equally informative, contingencies dominate processing. However, when mostly incongruent stimuli are uninformative this is no longer the case. For instance, if a color word is presented equally often in four colors, then the word is mostly incongruent (i.e., 75% incongruent), but it is not predictive of what to respond (i.e., each of the four responses are equiprobable). According to those authors, weakening the predictiveness of the distracters in this way impairs the contingency mechanism and allows conflict adaptation to play a role. In other words, they argue that conflict adaptation can occur, but only when contingency learning does not “steal the show.” In support of this, they found that when mostly incongruent stimuli were as predictive as mostly congruent stimuli, the data fit the predictions of the contingency account. However, when mostly incongruent stimuli were unpredictive and mostly congruent stimuli were (still) predictive, they found a different pattern of results. Specifically, they found mostly interference-driven effects in this scenario, with large impairments of incongruent items in the mostly congruent relative to mostly incongruent condition. In contrast, little differences were observed for congruent items. This is not what the contingency account should predict, especially since the only contingencies present in the task were for *congruent* items. Such a pattern is seemingly more consistent with the conflict adaptation account.

Results such as those in Bugg and Hutchison (2013) are thus quite interesting, because they suggest that conflict adaptation might indeed exist independent of contingency learning biases. However, the key critique of the current article is that the modified task configuration used in such experiments adds an additional complexity to the task. The contingency account was originally proposed to explain the simple scenario in which some words were mostly correlated with the congruent response, whereas other words were mostly correlated with an incongruent response (Schmidt and Besner, 2008). The word is equally informative in both scenarios. Generally speaking, when these same criteria were met in subsequent experiments, evidence for an exclusively contingency-driven account of the data remained compelling (e.g., Bugg and Hutchison, 2013; Schmidt, 2013b). Comparing a *contingent* mostly congruent condition to a *non-contingent* mostly incongruent condition might produce results that seem harder to interpret from a contingency learning perspective, but this might also be like comparing apples to oranges. It is known that a contingency-laden dimension must be attended in order to learn the correlation (e.g., Chun and Jiang, 2001). More importantly, it is also know that when a contingency is detected for a given distracting stimulus, attention is *attracted* to this stimulus (e.g., Chun and Jiang, 1998; Cosman and Vecera, 2014). For instance, Cosman and Vecera presented participants with a red or green cue on the left or right of the screen, followed by two letters in the two possible cue locations. One of the letters was a target, which participants identified, and the other not. Each letter was presented in either red or green. Neither the cue location nor the cue color predicted the color, location, or identity of the target. However, targets were presented most often in one color (e.g., red). As typically observed, responses were faster when the cue location matched the target location, indicating attentional capture of the cue. Most importantly, this attentional capture effect was larger when the cue was the color that the targets were typically presented in. This indicates that a contingent stimulus (e.g., red color) captures attention.

The notion that contingent stimuli attract attention only stands to reason: predictive stimuli in our environment are attended because they can help guide our behavior (see also, Hutcheon and Spieler, 2014). Thus, the suggestion here is that a correlated mostly congruent distracting word will attract more attention than an uncorrelated mostly incongruent one in experiments such as those of Bugg and Hutchison (2013). This is because the distracter is informative of the response in the former condition (i.e., contingent), but not in the latter condition. Thus, changes in attention across these two conditions will indeed lead to larger congruency effects in the mostly congruent condition. Specifically, attending to a mostly congruent word will have a large impairment on incongruent trials, due to an increase in interference. This explains the large impairments of incongruent trials in the (predictive) mostly congruent condition relative to the (non-predictive) mostly incongruent condition observed by Bugg and Hutchison (2013). Indeed, the expected results are the same as those for the conflict adaptation account, because both accounts predict that the congruency effect is modulated by attentional differences. Of course, the difference is what drives those attentional differences: contingencies or conflict.

Note again that differences in informativeness between mostly congruent and mostly incongruent stimuli are not always present in PC experiments (e.g., Jacoby et al., 2003). If words in the mostly incongruent condition are just as predictive of what response to make as in the mostly congruent condition, then informativeness is equated. Thus, attention would not vary across conditions. When Bugg and Hutchison (2013) removed this equality in informativeness between mostly congruent and mostly incongruent stimuli, however, the prior work on contingency learning discussed above suggests that attentional differences should become relevant. Note that this proposed attentional variation has nothing to do with an adaptation to conflict, however. The proposal is not that attention is pulled away from conflicting stimuli. Instead, the suggestion is that attention is *attracted* to stimuli that provide predictive information. Indeed, in the particular case of a PC task, a contingent word is also a viable cue for responding. Thus, attention may indeed be relevant, but conflict might not be.

Future work on this topic will need to focus not only on contingency confounds, simply speaking, but also on the overall informativeness of each stimulus. These considerations alone probably do not explain the entire range of data in the field (for a review of several others, see Schmidt, 2013a), but task regularities that allow the possibility for learning conflict-unrelated information do muddy the interpretation of any observed effects. The present analysis does not, however, only serve to question the interpretability of previously-published results. It is also hoped that this article might inspire future research on the potential role of attentional capture of contingent information in various conflict tasks. For instance, future research might attempt to assess stimulus informativeness biases independent of conflict. Informative stimuli might thus be shown to produce larger congruency effects, even if dissociated from PC. Complimentarily, one might attempt to test the notion of Bugg and Hutchison (2013) that conflict adaptation is observable when contingencies are absent or weak by constructing a situation in which mostly congruent and mostly incongruent stimuli are equally (un)informative. Disentangling these two accounts will probably be challenging, but would be fruitful if possible.

## Author contributions

James R. Schmidt is the sole author and is a postdoctoral researcher of the Research Foundation—Flanders (FWO—Vlaanderen).

### Conflict of interest statement

The author declares that the research was conducted in the absence of any commercial or financial relationships that could be construed as a potential conflict of interest.
